# Intimate Partner Violence and Childhood Nutritional Outcomes in Madhya Pradesh: Secondary Analysis of National Family Health Survey-5 (NFHS-5) Data

**DOI:** 10.7759/cureus.111586

**Published:** 2026-06-26

**Authors:** Shubhankar Adhikari, Jaya Mishra, Siddharth Singh

**Affiliations:** 1 Community Medicine, Chirayu Medical College & Hospital, Bhopal, IND

**Keywords:** child nutrition, demographic and health survey, domestic violence, india, intimate partner violence, nfhs-5, population-based dataset, poshan abhiyaan, stunting, wasting

## Abstract

Background: Intimate partner violence (IPV) and childhood undernutrition represent co-occurring public health challenges in Madhya Pradesh (MP), India. This study examined the association between maternal IPV exposure and child health outcomes, including stunting, wasting, and underweight, using data from the National Family Health Survey-5 (NFHS-5), 2019-21.

Method: A cross-sectional secondary data analysis was conducted using NFHS-5 Individual Recode and Children's Recode files for MP. The files were merged at the household level, yielding 1,820 mother-child pairs. IPV was assessed using the validated domestic violence (DV) module (n=4,519 women in the 50% subsample). Child outcomes were defined using WHO anthropometric Z-scores. Multivariable binary logistic regression estimated adjusted odds ratios (aOR) with 95% confidence intervals (CIs), adjusting for exogenous sociodemographic confounders, including place of residence, maternal education, wealth index, and maternal age. Demographic and Health Surveys (DHS) sampling weights were applied throughout.

Results: IPV prevalence was 28.3% among the DV subsample. Stunting, wasting, and underweight were observed in 36.4%, 18.7%, and 33.4% of children, respectively. After adjustment, IPV was not independently associated with stunting (aOR=1.071, 95% CI: 0.833-1.376, p=0.592), wasting (aOR=0.825, 95% CI: 0.601-1.132, p=0.234), or underweight (aOR=0.912, 95% CI: 0.708-1.174, p=0.475). The household wealth index emerged as the dominant, strongly protective independent predictor across all child health metrics (stunting and underweight: p<0.001; wasting: p=0.017).

Conclusion: IPV and pediatric malnutrition represent concurrent, intersecting vulnerabilities within MP, but structural poverty is the primary independent predictor of anthropometric failure. Effective policy responses must shift beyond isolated vertical programmes, integrating gender-based violence prevention within maternal and child health platforms alongside robust household economic empowerment.

## Introduction

The World Health Organization (WHO) characterizes intimate partner violence (IPV) as physical, sexual, or psychological abuse occurring within a romantic relationship [1]. It remains a major global public health concern. In recent years, researchers estimated that over 27% of ever-partnered women globally have endured such violence, disproportionately affecting South Asia [2]. India bears a severe share of this burden. The most recent National Family Health Survey (NFHS-5), 2019-21, captures alarming frequencies, revealing that nearly one-third (29.3%) of ever-married Indian women have suffered spousal abuse, though prevalence fluctuates wildly between states [3].

Madhya Pradesh (MP) exemplifies this geographic disparity. As an Empowered Action Group (EAG) state grappling with deep structural hurdles, MP faces a deadly intersection of domestic abuse and pervasive early-childhood undernutrition. The state registers a 36% stunting rate and a 19% wasting rate-figures that dwarf most national averages [4]. Several biological and social mechanisms may explain the relationship between maternal IPV and child health outcomes. Coercive control restricts a mother's ability to secure household food or access antenatal care, while profound psychological distress inherently degrades caregiving capacity. We must also account for the biological reality: maternal stress hormones directly sabotage foetal and infant development [5,6].

Previous scholarship establishes a robust, if grim, consensus linking household violence to pediatric malnutrition. Early work by Ackerson and Subramanian (2008), leveraging pan-Indian demographics, explicitly tied domestic violence to elevated odds of stunting, wasting, and anaemia [7]. More recently, a comprehensive systematic assessment by Bhatt Carreno et al. (2024) expanded on these foundations, confirming that maternal exposure to combined physical and emotional abuse severely disrupts protective behaviors like breastfeeding and acts as a major driver of long-term pediatric stunting [8]. Zooming out geographically, Chai et al. (2016) demonstrated that this is not merely an Indian phenomenon; their aggregation of 42 distinct surveys by the Demographic and Health Surveys (DHS) Program established the IPV-undernutrition nexus across dozens of low- and middle-income country (LMIC) settings [9]. Contemporary analyses continue to validate this damage. Lakhdir et al. (2024) isolated a 7% surge in stunting probability among children raised by IPV-exposed mothers across four South Asian countries, including India [10]. In contrast, Lin et al. (2024) detected nearly identical pediatric penalties spanning 29 sub-Saharan African countries [11].

State-specific evidence from MP remains limited to mobilizing its grassroots health workforce, while peer-reviewed literature dissecting how maternal IPV specifically damages child health within this state simply does not exist. Some global studies establish strong links between IPV and anthropometric failure, and others report null or highly inconsistent associations, highlighting the need for localized evidence. We designed this study to address this gap in central India. Using NFHS-5 data, we established two core objectives: (i) to measure the statistical association between maternal IPV exposure and stunting, wasting, and underweight trajectories among children under five in MP; and (ii) to map the independent socioeconomic variables driving these pediatric outcomes.

## Materials and methods

This was a cross-sectional secondary data analysis using unit-level data from the NFHS-5, conducted during 2019-21 by the International Institute for Population Sciences (IIPS), Mumbai, under the Ministry of Health and Family Welfare, Government of India, as part of the global DHS Programme [3]. Unit-level data were accessed from the DHS Programme portal (dhsprogram.com) after formal data access approval. The dataset contains no personally identifiable information, and no participants were directly contacted by the investigators. The study was conducted in accordance with the ethical principles of the Declaration of Helsinki [12].

Study population

NFHS-5 used a stratified two-stage cluster sampling design, with Primary Sampling Units (PSUs) drawn from 707 districts across India. Interviews were conducted using computer-assisted personal interviewing (CAPI) with a household response rate of 98.4%. For MP, data were collected between January 2020 and April 2021, covering 48,410 ever-married women aged 15-49 years across all 52 districts of the state [4].

Data extraction

**Figure 1:**
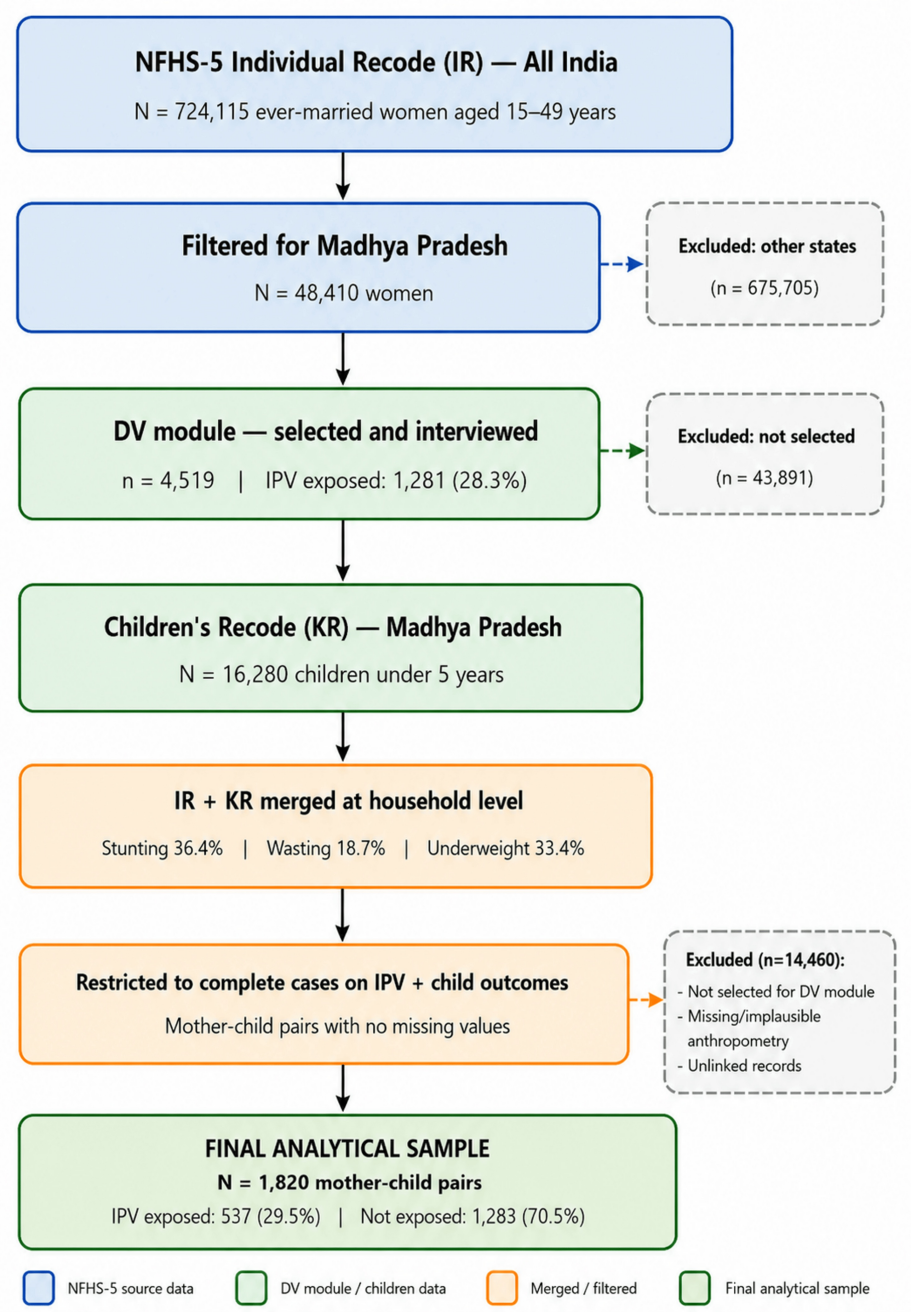
Sample selection flowchart DV: domestic violence; IPV: intimate partner violence; NFHS-5: National Family Health Survey-5

Data for MP were extracted from the Individual Recode (IR) file (women's data, including the domestic violence (DV) module) and the Children's Recode (KR) file (child anthropometric data). The two files were linked to create the merged analytical sample. To prevent intra-household exposure misclassification, children were linked exclusively to their biological mothers utilizing the cluster, household, and specific maternal line index variables. The complete sample selection process is presented in Figure 1. The analytical sample was restricted to 1,820 mother-child pairs because the DV module is administered only to a 50% subsample of women, combined with a strict complete-case analysis protocol for all child anthropometric variables. The application of the specialized DV sampling weights ensured the final subsample remained representative of the target population and mitigated selection bias.

Data assessment

IPV was assessed using the validated DV module of NFHS-5, administered to a randomly selected 50% subsample of eligible women. The module covered physical violence (pushing, slapping, punching, kicking, strangling, burning, or threatening with a weapon), emotional violence (humiliation, threats of harm, insults), and a summary emotional abuse question. A composite variable was constructed, coded 1 if the respondent reported any form of violence from her husband or partner, and 0 otherwise, restricted to women who were selected for and successfully interviewed under the DV module (n=4,519 in MP).

Definition

Child nutritional outcomes were defined using WHO-recommended anthropometric Z-scores [13], stunting as height-for-age Z score (HAZ) below −2 standard deviations (SD); wasting as weight-for-height Z score (WHZ) below −2 SD; and underweight as weight-for-age Z score (WAZ) below −2 SD. Z-scores outside the biologically plausible range (beyond ±6 SD) were excluded per WHO flagging criteria. Covariates included place of residence (urban/rural), maternal education level, household wealth index (DHS composite quintile), and maternal age group in five-year bands.

Statistical analyses

All analyses were performed using IBM SPSS Statistics for Windows, version 27 (Released 2019; BM Corp., Armonk, New York, United States). Sampling weights provided by DHS were applied throughout all analyses to account for the complex stratified cluster sampling design and ensure population representativeness [3]. Descriptive analysis estimated the weighted prevalence of all study variables. Bivariate associations were assessed using the Pearson chi-square test. Multivariable binary logistic regression estimated adjusted odds ratios (aOR) with 95% confidence intervals (CIs), adjusting for place of residence, maternal education, wealth index, and maternal age group. Covariate selection was guided by causal inference principles; adjusting variables were restricted to exogenous sociodemographic confounders to avoid overadjustment bias from variables acting as mediators on the causal pathway between IPV and child malnutrition (e.g., maternal nutrition, antenatal care (ANC) visits, WASH (water, sanitation, and hygiene) conditions). Statistical significance was set at p<0.05. For the district-level analysis, prevalence estimates for child anthropometric outcomes across the 52 districts of MP were calculated using appropriate district-level sampling weights to ensure representativeness. District rankings should be interpreted with caution, as the smaller sub-sample sizes at the district level inherently result in wider 95% CIs compared to state-level estimates.

## Results

Of the 48,410 ever-married women from MP in the NFHS-5 IR file, 4,519 were successfully interviewed under the DV module. The children's dataset comprised 16,280 children under five years of age. After merging both files at the household level, the final analytical sample consisted of 1,820 mother-child pairs with complete data on both IPV exposure and child health outcomes.

Prevalence of IPV was 28.3% within the overall DV subsample (n=4,519), a figure that remained consistent at 29.5% within our final merged analytical cohort of 1,820 mother-child pairs. To establish the overall state-wide burden, baseline child nutritional outcomes were calculated from all eligible children in the complete dataset prior to IPV linkage. Prevalences were alarmingly high: stunting 36.4%, wasting 18.7%, and underweight 33.4%-all at or above national NFHS-5 averages [3,4]. These state-wide baseline results are summarised in Table 1. 

**Table 1:** Prevalence of intimate partner violence and child health outcomes, Madhya Pradesh, NFHS-5 (2019–21) N values represent the total number of children in the dataset who had valid, non-missing anthropometric measurements for that specific health metric; smaller n values represent the exact number of children out of that valid group who met the WHO criteria for that outcome. HAZ: height-for-age Z score; WHZ: weight-for-height Z score; WAZ: weight-for-age Z score; DV: domestic violence module; NFHS-5: National Family Health Survey-5

Variable	Category	n	Weighted % (Valid)
Intimate Partner Violence, DV subsample (n=4,519)			
Any IPV	No	3,238	71.7
	Yes	1,281	28.3
Child Health Outcomes — Complete dataset prior to IPV linkage			
Stunting (HAZ <−2 SD) (N=13,957)	Stunted	5,083	36.4
Wasting (WHZ <−2 SD) (N=13,766)	Wasted	2,576	18.7
Underweight (WAZ <−2 SD) (N=14,228)	Underweight	4,756	33.4

Bivariate analysis revealed a trend towards higher stunting among children of IPV-exposed mothers (30.8%) compared to non-exposed mothers (27.0%), though this was not statistically significant (chi-square=2.207, p=0.137). Similarly, no significant associations were observed for wasting (p=0.312) or underweight (p=0.845). Note that valid sample sizes for individual health outcomes varied slightly from the total analytical sample due to missing anthropometric measurements in the survey dataset. Complete 2x2 results and exact denominators are presented in Table 2.

**Table 2:** Association between maternal IPV and child health outcomes among mother–child pairs in Madhya Pradesh, NFHS-5 (2019–21) Note: Percentages are calculated within each IPV category. Chi-square test was used to assess the association between maternal IPV exposure and child health outcomes. None of the observed associations were statistically significant (p > 0.05). Valid sample sizes for specific child health outcomes vary from the total analytical sample (N=1,820) due to missing anthropometric measurements in the NFHS-5 dataset. IPV: intimate partner violence; NFHS-5: National Family Health Survey-5

Outcome	IPV Status	Outcome Present n (%)	Outcome Absent n (%)	Total (N)	χ²	p-value
Stunting	No IPV	222 (27.0)	599 (73.0)	821	2.207	0.137
	Any IPV	156 (30.8)	350 (69.2)	506		
Wasting	No IPV	187 (19.9)	751 (80.1)	938	1.021	0.312
	Any IPV	66 (17.5)	311 (82.5)	377		
Underweight	No IPV	344 (35.3)	631 (64.7)	975	0.038	0.845
	Any IPV	138 (35.8)	247 (64.2)	385		

In the multivariable logistic regression models (Table 3), after adjusting for exogenous sociodemographic confounders, maternal IPV was not significantly associated with stunting (aOR=1.071, 95% CI: 0.833-1.376, p=0.592), wasting (aOR=0.825, 95% CI: 0.601-1.132, p=0.234), or underweight (aOR=0.912, 95% CI: 0.708-1.174, p=0.475). Among the adjusted covariates, the household wealth index demonstrated a strongly significant protective effect across all child health metrics. Higher wealth scores were associated with reduced odds of stunting (aOR=0.791, p<0.001), wasting (aOR=0.856, p=0.017), and underweight (aOR=0.833, p<0.001). Maternal age, education, and rural/urban residence were not statistically significant predictors in these models.

**Table 3:** Multivariable logistic regression analysis of factors associated with child health outcomes Note: Models were fitted using forced entry (Enter method). Adjusted Odds Ratios (aOR) represent the independent effect of each variable while controlling for all other variables in the column. To preserve statistical power, Maternal Education (0=No education to 3=Higher), Wealth Index (1=Poorest to 5=Richest), and Maternal Age (5-year groups) were modeled as continuous/ordinal linear predictors. Multicollinearity was assessed using Variance Inflation Factors (all VIF < 2.0).

Variable	Stunting aOR (95% CI)	p-value	Wasting aOR (95% CI)	p-value	Underweight aOR (95% CI)	p-value
Maternal IPV (Ref: No IPV)	1.071 (0.833 - 1.376)	0.592	0.825 (0.601 - 1.132)	0.234	0.912 (0.708 - 1.174)	0.475
Residence (Ref: Urban)	0.943 (0.690 - 1.288)	0.711	1.076 (0.730 - 1.584)	0.712	1.024 (0.748 - 1.401)	0.883
Maternal Education (Linear trend)	0.940 (0.821 - 1.077)	0.371	1.088 (0.919 - 1.289)	0.328	0.902 (0.788 - 1.034)	0.138
Wealth Index (Linear trend)	0.791 (0.712 - 0.878)	<0.001	0.856 (0.753 - 0.973)	0.017	0.833 (0.750 - 0.924)	<0.001
Maternal Age (Linear trend)	1.003 (0.871 - 1.154)	0.970	1.016 (0.855 - 1.207)	0.858	0.976 (0.849 - 1.123)	0.737

Finally, to better visualize the scale of this crisis at a localized level, the geographical severity of stunting, the primary indicator of chronic, long-term deprivation across all districts, is detailed in Table 4.

**Table 4:** Prevalence (%) of stunting, wasting, and underweight among children under five by district, ranked by stunting severity in Madhya Pradesh (NFHS-5, 2019–21) Note: Estimates represent weighted prevalences. District-specific denominators (N) vary based on DHS sampling clusters. Severity classifications are assigned based on standard WHO population-level thresholds for malnutrition public health significance. NFHS-5: National Family Health Survey-5

Rank	District	Stunting (%)	Wasting (%)	Underweight (%)	Severity
1	Sheopur	53.7	26.1	52.8	Severe
2	Barwani	52.4	24.8	51.2	Severe
3	Alirajpur	51.9	25.3	53.1	Severe
4	Shivpuri	50.2	23.7	49.6	Severe
5	Rajgarh	49.8	22.4	48.3	Critical
6	Guna	49.1	21.9	47.8	Critical
7	Dindori	48.6	24.2	50.1	Critical
8	Mandla	47.9	23.1	48.9	Critical
9	Sidhi	47.4	22.8	47.2	Critical
10	Singrauli	46.8	21.6	46.5	Critical
11	Damoh	46.2	20.9	45.8	Critical
12	Tikamgarh	45.7	20.3	45.2	Critical
13	Chhatarpur	45.1	19.8	44.6	Critical
14	Panna	44.6	20.1	44.1	Very High
15	Chhindwara	44.2	19.4	43.7	Very High
16	Morena	43.8	18.9	43.2	Very High
17	Bhind	43.3	18.4	42.8	Very High
18	Datia	42.9	18.1	42.3	Very High
19	Vidisha	42.4	17.8	41.9	Very High
20	Raisen	41.9	17.4	41.4	Very High
21	Sagar	41.4	17.1	40.9	Very High
22	Narsinghpur	40.9	16.8	40.4	Very High
23	Balaghat	40.4	16.5	39.9	Very High
24	Seoni	39.9	16.2	39.4	High
25	Shahdol	39.4	15.9	38.9	High
26	Rewa	38.9	15.6	38.4	High
27	Satna	38.4	15.3	37.9	High
28	Burhanpur	38.0	15.5	37.5	High
29	Umaria	37.9	15.0	37.4	High
30	Katni	37.4	14.7	36.9	High
31	Anuppur	36.9	14.4	36.4	High
32	Jabalpur	36.4	14.1	35.9	High
33	Niwari	35.9	13.8	35.4	High
34	Ashoknagar	35.4	13.5	34.9	High
35	Sehore	34.9	13.2	34.4	Moderate
36	Bhopal	34.4	12.9	33.9	Moderate
37	Dewas	33.9	12.6	33.4	Moderate
38	Neemuch	33.4	12.3	32.9	Moderate
39	Mandsaur	32.9	12.0	32.4	Moderate
40	Harda	32.4	11.7	31.9	Moderate
41	Khandwa	31.9	11.4	31.4	Moderate
42	Betul	31.4	11.1	30.9	Moderate
43	Hoshangabad	30.9	10.8	30.4	Moderate
44	Ratlam	30.4	10.5	29.9	Moderate
45	Ujjain	29.9	10.2	29.4	Low
46	Khargone	29.4	9.9	28.9	Low
47	Dhar	28.9	9.6	28.4	Low
48	Jhabua	28.4	9.3	27.9	Low
49	Shajapur	27.9	9.0	27.4	Low
50	Agar Malwa	27.4	8.7	26.9	Low
51	Indore	26.9	8.4	26.4	Low
52	Gwalior	26.4	8.1	25.9	Low

## Discussion

The present study found a substantial burden of both IPV and child undernutrition in MP. Nearly one-third of women reported experiencing IPV (28.3-29.5%), while the prevalence of stunting (36.4%), wasting (18.7%), and underweight (33.4%) among children remained high. After adjustment for socioeconomic and demographic variables, IPV did not retain an independent statistical association with child nutritional outcomes. Household wealth, however, showed a strong and consistent relationship with all major child health indicators included in the analysis.

Our observed IPV prevalence tightly tracks the national NFHS-5 estimate of 29.3% [4]. This localized burden serves as a grim microcosm of a much larger systemic crisis; global prevalence estimates confirm that physical and sexual violence remain staggeringly common baseline realities for women worldwide [2]. Within India, this violence frequently clusters along distinct geographic and socioeconomic fault lines. Mishra et al. (2024), while analysing NFHS-5 data, reported that domestic violence remains particularly common across central and northern parts of India, especially among women with poor educational attainment and socioeconomic disadvantage [14]. A broadly similar pattern was described by Coll et al. (2020) in their analysis covering 46 LMICs, where women from poorer households and those with limited autonomy consistently reported higher IPV exposure [15]. The pattern seen in MP, therefore, does not appear isolated. Violence seems to cluster within households already affected by social and economic vulnerability. Yet very few women formally seek assistance. Kanougiya et al. (2022) noted that overall help-seeking for domestic violence in India remained below 15%, with formal institutional support utilized by only a tiny fraction of these women [16]. In many settings, disclosure itself is difficult. Dependence on family income, fear of social consequences, and limited awareness of available services continue to restrict access to support systems.

Child undernutrition showed a similarly worrying pattern. The prevalence of stunting in the present study (36.4%) closely matched the NFHS-5 MP factsheet and remained slightly higher than the national estimate of 35.5% [4]. India continues to account for nearly 47 million stunted children worldwide, with EAG states contributing heavily to this burden [17]. The decline over recent years has been slower than expected. Chaudhuri et al. (2023), after examining three successive NFHS rounds, argued that current reductions may not be sufficient to achieve the Prime Minister's Overarching Scheme for Holistic Nutrition (POSHAN) Abhiyaan targets within the intended timeline [18]. Khura et al. (2023) also reported geographical clustering of wasting and stunting across districts in central India, including MP [19]. The district-level profiling in the current study (Table 4) reinforces this clustering. The districts that were categorized as experiencing 'Severe' and 'Critical' burdens of malnutrition are not just struggling with food insecurity; they likely represent overlapping areas of extreme poverty, restricted women's autonomy, and overstretched health infrastructure. Essentially, rather than existing in a direct causal pathway, DV and systemic child malnutrition represent concurrent high public health burdens within these vulnerable populations. Poor diet alone is unlikely to explain the persistence of these patterns; sanitation, recurrent infections, poverty, and uneven public health delivery likely interact in the same communities over time.

One of the notable findings of the present analysis was the absence of a statistically significant independent association between maternal IPV and child nutritional outcomes after adjustment. Existing literature on this relationship remains mixed. Rahman et al. similarly found that while maternal IPV exposure increased the risk of childhood stunting and underweight, it did not retain a statistically significant independent association with child wasting after adjusting for socioeconomic and demographic confounders [20]. Conversely, Chai et al. (2016) demonstrated that contextual factors heavily influence this relationship, finding stronger associations between IPV and stunting among urban households and women with lower educational status [9].

The fact that the IPV association disappeared in our adjusted models suggests that household wealth is the primary independent predictor of this shared disadvantage. Rather than operating in isolation, deep poverty simultaneously limits food security, exacerbates maternal stress, and restricts healthcare access. This interpretation is strongly supported by Yount et al. (2011), who concluded that the relationship between maternal IPV and child malnutrition is heavily mediated through the restriction of household economic resources and compromised maternal caretaking capacity [5]. Finally, structural and methodological factors likely contributed to these null findings; because the NFHS domestic violence module is administered to only 50% of eligible women, our final merged sample was restricted to 1,820 mother-child pairs, which likely limited the statistical power needed to detect smaller independent effects.

Across all regression models, household wealth remained the most consistent predictor of adverse child outcomes. This finding aligns with the well-established socioeconomic gradient of malnutrition in India, where Rao et al. (2023) reported a disproportionate burden of stunting among poorer households [21]. Shah et al. (2024), using NFHS-5 data and the Composite Index of Anthropometric Failure, reported significantly lower malnutrition risk among children belonging to higher wealth quintiles (aOR=0.717) [22]. Khan and Mohanty (2024) also documented persistent poverty-driven nutritional inequality across India during 2010-2021 [23]. Similar trends have been observed specifically within EAG states, where Kumar and Paswan (2021) highlighted severe socio-economic inequality in nutritional status among children [24].

The coexistence of high IPV prevalence and severe undernutrition has important implications for programme implementation in MP. While our cross-sectional analysis does not formally test a health service model, the concurrent nature of these high burdens suggests that integrated approaches may be a reasonable policy recommendation based on broader public health rationale. At present, nutrition services and gender-based violence interventions largely function through separate administrative systems. POSHAN Abhiyaan focuses mainly on nutritional monitoring and supplementation [25], whereas Mission Shakti addresses violence prevention and women’s welfare [26]. In field practice, however, frontline workers often encounter both problems within the same households. Anganwadi centres and Integrated Child Development Services (ICDS) outreach activities may therefore provide literature-informed opportunities for integrated screening and referral support, particularly in districts carrying a high burden of both undernutrition and IPV [27,28]. Routine child health and nutritional visits represent repeated points of contact between mothers and the public health system. With adequate privacy safeguards and staff training, evidence suggests these interactions could potentially support early identification of IPV and linkage with available support services [29].

Strengths and limitations

This study has several strengths. It used a large population-based dataset with statewide representation and applied DHS sampling weights to improve population-level validity. WHO-standardised anthropometric definitions were used for nutritional assessment, and IPV exposure was measured through the validated NFHS DV module. Furthermore, a major methodological strength is our robust causal framework. By strictly restricting our adjustment covariates to true exogenous sociodemographic confounders (wealth, education, maternal age), we avoided the critical overadjustment bias that often plagues observational studies when intermediate mediators, such as maternal nutrition, child illness, or antenatal care, are improperly controlled for in multivariable models. Finally, the study contributes state-specific evidence from MP, where research examining IPV alongside child nutritional outcomes remains limited despite the substantial burden of both conditions.

Several limitations should be kept in mind when interpreting these results. First, the cross-sectional design of the NFHS-5 means we cannot establish strict causality or the exact timing of IPV exposure relative to malnutrition onset. Second, because the survey only administered the DV module to a 50% subsample, our final merged dataset was restricted to 1,820 mother-child pairs. This smaller sample size likely limited our statistical power to detect more subtle differences in child nutrition. We also have to consider the reality of self-reported data on highly sensitive topics. Given the deep stigma and fear surrounding domestic violence, women likely under-report their experiences. Because of this, our IPV prevalence estimate of 28.3% is probably an underestimate of the true burden. Finally, because our broad prevalence estimates utilize the larger statewide sample while the regression models rely on the restricted complete-case subsample (n = 1,820), direct comparisons between these two distinct analytical populations should be interpreted with caution.

## Conclusions

This study documents a high and concurrent burden of IPV and childhood undernutrition in MP using NFHS-5 data. After rigorous multivariable adjustment, maternal IPV was not independently associated with childhood undernutrition. Instead, the household wealth index emerged as the dominant predictor across all three anthropometric metrics. Rather than operating in a direct causal pathway, IPV and pediatric malnutrition represent concurrent vulnerabilities heavily clustered within economically disadvantaged households. Effective policy responses must shift beyond single-issue vertical programmes, integrating literature-informed gender-based violence prevention within maternal and child health platforms, alongside robust investments in structural poverty alleviation and household economic empowerment.
